# Evidence for an Age-Related Positivity Effect in Metacognitive Confidence Judgments

**DOI:** 10.1093/geroni/igaa057.1170

**Published:** 2020-12-16

**Authors:** Edie Sanders, Jane Berry

**Affiliations:** 1 Florida State University, Tallahassee, Florida, United States; 2 University of Richmond, Richmond, Virginia, United States

## Abstract

We examined age differences in metacognitive monitoring of emotionally-valenced stimuli. If older adults (OAs) are more focused on emotionally meaningful goals in late life (Carstensen, 2006), then they should demonstrate attentional and memory biases for positive stimuli over neutral and negative stimuli and, arguably, these cognitive biases should be reflected in their metacognitive judgments of learning. Judgments of learning (JOLs) for memory of positive, negative, and neutral words were collected. Younger adults (YAs) aged 18-23 years and OAs aged 65-90 years (N = 85) studied words in each valence category and made immediate JOLs, followed by a two-alternative forced choice (2AFC) recognition memory task. Analyses of JOLs revealed evidence for a positivity effect (Mather & Carstensen, 2005) in metacognitive confidence for OAs and an emotional salience effect in YAs (Tauber & Dunlosky, 2012; Zimmerman & Kelley, 2010). Predictably, YAs recognized more words than OAs, but valence did not affect number of words recognized and valence did not moderate age differences in recognition memory (p = .055). Memory monitoring as measured by resolution accuracy was equivalent in YAs and OAs (Hertzog & Dunlosky, 2011). Positive affect was higher and negative affect was lower in OAs relative to YAs (Gallant, Spaniol, & Yang, 2019), lending additional evidence to an orientation toward the positive in older adulthood. These results are novel in that they demonstrate an age-related positivity effect that extends beyond the domains of memory and emotion to the domain of metacognitive aging. Discussion will focus on theoretical, methodological, and applied implications.

